# Diabetic Dead-in-Bed Syndrome: A Possible Link to a Cardiac Ion Channelopathy

**DOI:** 10.1155/2014/647252

**Published:** 2014-02-19

**Authors:** Jonathan R. Skinner, Renate Marquis-Nicholson, Alix Luangpraseuth, Rick Cutfield, Jackie Crawford, Donald R. Love

**Affiliations:** ^1^Cardiac Inherited Disease Group, Auckland City Hospital, Auckland 1148, New Zealand; ^2^Green Lane Paediatric and Congenital Cardiac Services, Starship Children's Hospital, Auckland 1148, New Zealand; ^3^Department of Child Health, University of Auckland, Private Bag 92019, Auckland 1142, New Zealand; ^4^Diagnostic Genetics, LabPlus, Auckland City Hospital, Auckland 1148, New Zealand; ^5^Department of Pathology, University of Melbourne, Parkville, VIC 3010, Australia; ^6^School of Biological Sciences, University of Auckland, Private Bag 92019, Auckland 1142, New Zealand; ^7^INRA, UMR 1198, Biologie du Développement et Reproduction, 78350 Jouy-en-Josas, France; ^8^Diabetes Service, North Shore Hospital, Takapuna, Auckland 0622, New Zealand

## Abstract

Sudden unexpected nocturnal death among patients with diabetes occurs approximately ten times more commonly than in the general population. Malignant ventricular arrhythmia due to Brugada syndrome has been postulated as a cause, since a glucose-insulin bolus can unmask the Brugada electrocardiographic signature in genetically predisposed individuals. In this report we present a 16-year-old male with insulin-dependent diabetes who died suddenly at night. His diabetes had been well controlled, without significant hypoglycaemia. At autopsy, he had a full stomach and a glucose level of 7 mmol/L in vitreous humor, excluding hypoglycaemia. Genetic analysis of autopsy DNA revealed a missense mutation, c.370A>G (p.Ile124Val), in the *GPD1L* gene. A parent carried the same mutation and has QT prolongation. Mutations in this gene have been linked to Brugada syndrome and sudden infant death. The patient may have died from a ventricular arrhythmia, secondary to occult Brugada syndrome, triggered by a full stomach and insulin. The data suggest that molecular autopsies are warranted to investigate other cases of the diabetic dead-in-bed syndrome.

## 1. Introduction

Sudden unexpected nocturnal death among patients with diabetes is greatly feared and poorly understood, occurring approximately ten times more commonly than in the general population [1]. The “dead-in-bed” syndrome, by definition, has a negative autopsy and accounts for up to 6% of all deaths in type I diabetics under the age of 40 years [2]. Hypoglycaemia has been put forward as the most likely explanation but has been excluded in some cases [2–4]. The possibility that a cardiac ion channelopathy such as long QT syndrome or Brugada syndrome may cause death through a malignant arrhythmia in individuals with diabetes has been considered but never proven [5].

We report the postmortem molecular genetic investigation of a 16-year-old boy with type 1 diabetes who died in his sleep. His blood glucose had been well-controlled; he had a full stomach at autopsy and a glucose level in vitreous humor of 7 mmol/L. These features argue strongly against hypoglycaemia. Molecular genetic investigation revealed a missense mutation (p.Ile124Val) in the *GPD1L* gene. GPD1-L catalyses the conversion of sn-glycerol 3-phosphate to glycerone phosphate and binds to the SCN5A ion channel protein; mutations in the *GPD1L* gene have been linked to Brugada syndrome and to sudden infant death syndrome [6–8].

## 2. Case History

The boy was diagnosed as having type 1 diabetes at the age of 15 years (nine months prior to his death) presenting with mild diabetic ketoacidosis following a nine-month history of polydipsia and polyuria. He was managed with insulin and at the time of his death was receiving four injections per day: insulin aspart three times daily (approximately 12 units at meals, varied by carbohydrate load and prevailing blood sugar level) and insulin glargine 30 units at bed time. Three months prior to his death, his HbA1c level was 7.3% (56 mmol/mL). He was fully compliant with therapy and home glucose monitoring, and he remained physically active and fit. Review of his monitoring book and history revealed no episodes of severe hypoglycaemia or obvious nocturnal hypoglycaemia. On the evening prior to his death he had been completely well, had a normal full meal, had his insulin, and went to bed as usual at approximately 9:30 p.m. He was found dead the following morning lying face down on his bed and could not be resuscitated. He had no previous history of syncope or seizure and never had cause to have any cardiac tests during his life.

At autopsy, there were no pathological findings to indicate a cause of death. Cardiac and cerebral examinations were normal. Significantly, however, he had a full stomach, suggesting that he died soon after his meal. A standard toxicology screen was negative. The glucose level in his vitreous humor excluded hypoglycaemia at 7 mmol/L (126 mg/dL) [9]. DNA was extracted and stored from a peripheral blood sample and the family was referred for cardiac genetic investigation as is routine in New Zealand for sudden unexplained deaths between one and 40 years of age [10].

## 3. Molecular Analysis of Postmortem Sample

**Figure 1:**
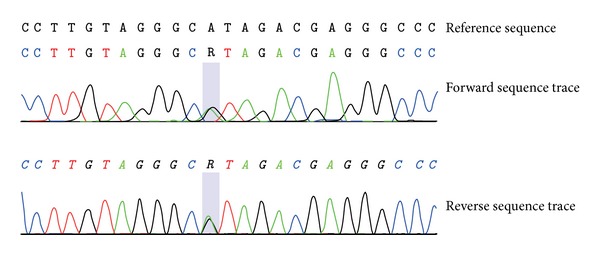
Sequence analysis of proband. Sequence electropherograms show the presence of the c.370A>G, p.Ile124Val (p.I124V), mutation in exon 4 of the *GPD1L* gene.

The coding regions for those genes linked to Long QT syndrome types 1, 2, 3, 5, 6, and 7 were amplified and subjected to capillary-based sequencing, as described earlier [11], but no mutations were identified. Amplification of the coding regions of the *GPD1L* gene (Refseq accession number NM_015141.3; primer designs based on [12]) and subsequent sequencing revealed a heterozygous missense mutation in exon 4: c.370A>G, p.Ile124Val (Figure 1).

## 4. Family Investigation

**Figure 2:**
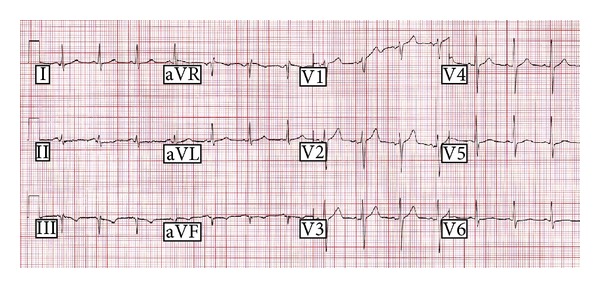
12-lead electrocardiagram of the deceased's father. The ST-T segment is unusual and very flat in lead II, such that determining the end of the T wave is difficult. In lead I, R-R is 690 ms, QT is 396 ms, and QTc is 477 ms. In lead V5, R-R is 711 ms, QT s 391 ms, and QTc is 464 ms; the T wave is of low amplitude with a slight double bump morphology.

There was no known history of sudden death in the family. Both parents and his only sibling were investigated. 12 lead ECGs were normal in the mother and sibling. Exercise testing and cardiac magnetic resonance imaging were normal in the mother. The father's ECG showed subtly abnormal repolarisation with a marginally prolonged QT interval; the heart-rate corrected QT interval (QTc) was 0.46 seconds (on no medication) (Figure 2). The maternal aunt had a history of syncope that sounded vasovagal in nature. Her ECG, exercise testing, and cardiac magnetic resonance imaging tests were normal.

After genetic counselling, DNA was obtained from the father, mother and sister and these were tested for the presence of the mutation c.370A>G, p.Ile124Val, in the *GPD1L* gene. The father carried the mutation, whereas the mother, and sibling did not. Pharmacological challenge to the cardiac sodium channel was performed on the father using intravenous ajmaline at 1 mg/kg over ten minutes. No evidence of Brugada signature was unmasked.

## 5. Discussion

This case suggests a cause of death in a patient with diabetes who died in bed with a negative autopsy. It demonstrates a possible link between dead-in-bed syndrome and Brugada syndrome, thereby supporting earlier work [13], albeit for a *GPD1L* gene variant of unclear clinical significance but with supportive *in vitro *evidence.

Cardiac ion channelopathies explain 20 to 40% of autopsy negative sudden death in 1–40-year-olds [10, 14–16]. Most such deaths in this age group occur at rest or during sleep, and these diagnoses are reached by cardiac/genetic investigation of either autopsy DNA or living family members. Mutations in the *GPD1L* gene were first reported in 2007 to be causative of sudden infant death and familial Brugada syndrome [6, 7]. GPD1L has a functional link with the cardiac sodium channel Na_v_1.5 [7], which is encoded for by the *SCN5A* gene. Mutations in the latter gene are the most common single cause of sudden infant death syndrome (SIDS) and familial Brugada syndrome. They also cause long QT syndrome type 3. Death is due to ventricular fibrillation or ventricular tachycardia.

Death during sleep, most commonly in young males, is typical in Brugada syndrome and Long QT type 3, both of which are caused by dysfunction of the cardiac sodium ion channel Na_v_1.5 [17]. Long QT syndrome and Brugada syndrome can occur with the same mutation, even in the same family with age, heart rate, fever, and other developmental or environmental influences playing a role (cardiac sodium channel overlap syndrome) [17].

In Brugada syndrome a diagnostic cardiac depolarization abnormality is seen on the 12-lead ECG with right-bundle branch block appearance in V1 and V2 associated with ST elevation [18]. In genetically predisposed children, fever is a potent trigger for malignant arrhythmias [19, 20].

There is some conflicting evidence regarding the pathogenicity of the mutation reported here. I124V was initially discovered in a 5-week-old infant who died suddenly and had a negative postmortem examination [7]. The mutation is in a highly conserved residue and, when coexpressed *in vitro* with SCN5A in heterologous HEK cells (Human Embryonic Kidney 293 cells), produces a significantly reduced sodium channel current [7]. The NCBI database records the variant as likely pathogenic (http://www.ncbi.nlm.nih.gov/clinvar/?term=rs72552293; entry dated August 18, 2011). In contrast, bioinformatic analysis of the mutation event at the protein level predicts a benign as well as a disease-causing effect, while three programmes predict no effect regarding splicing of the *GPD1L* gene transcript and one predicts an effect on SR protein binding sites (Table 1). SR proteins can influence splice site selection [21–25], but transcript analysis of the *GPD1L* gene has not been undertaken in the case reported here. The c.370A>G variant has been reported at frequencies of 0.14% and 0.11% in the 1000 Genomes project and GO-ESP databases, respectively. These data suggest that the variant is present in nominally unaffected individuals and so may be nonpathogenic.

It is interesting that the father, who carries the same mutation, had a negative ajmaline challenge. Ajmaline is a cardiac sodium channel blocker, and 75–80% of Brugada syndrome mutation carriers can be unmasked by it [26]. Variable clinical expression of these mutations is common, possibly due to the effect of common polymorphisms within the same gene or in *SCN5A* [27]. However, the father does have a marginally prolonged QT interval.

Supportive evidence for a link between the diabetic dead-in-bed syndrome and cardiac sodium channel dysfunction comes from a recent report from an adult male with diabetes [28]. He displayed a Brugada signature on his ECG during hyperglycaemic and hyperkalaemic ketoacidosis and his son also had Brugada syndrome. The authors postulated that the biochemical disturbances were to blame for transient cardiac sodium channel blockade in a genetically predisposed individual.

A large meal also unmasks the Brugada sign in genetically predisposed individuals. Ikeda et al. [29] showed that the “full stomach test” was positive in half of the subjects evaluated, and the appearance of the Brugada sign with a full stomach was more likely in those with a history of malignant arrhythmia. In addition, glucose infusion followed by insulin injection unmasks the Brugada sign [30, 31]. Thus, the full stomach found in our patient not only makes hypoglycaemia very unlikely but also supports the suggestion that he died from Brugada syndrome.

Another group has looked for genetic abnormalities in patients with diabetes who have died suddenly. Tu et al. [5] studied DNA retrospectively from 22 such cases and found no mutations in either the *SCN5A* gene or the *G6PC*, *PHOX2B*, and *CTGF* genes. The latter three genes are involved in gluconeogenesis/glycogenolysis, dysautonomia, and endothelial cell function, respectively; however, they did not analyze the *GPD1L *gene or any of the other Long QT genes. Further, they had to use DNA extracted from less robust sources such as paraffin embedded tissue blocks, where complete analysis is rarely possible.

The death of this 16-year-old boy presumably occurred due to a malignant ventricular arrhythmia caused by the combination of a genetic predisposition to occult cardiac Brugada syndrome and an electrolyte disturbance brought about by a full stomach and a high level of insulin. This case raises the possibility that some other young people with diabetes who have died unexpectedly at night will have done so due to a coexistent inherited arrhythmic predisposition. This might include long QT syndrome, for example, since hypoglycaemia is known to be potentially proarrhythmic in diabetes through QT interval prolongation and intracellular calcium overload [32].

Prevention of deaths in inherited arrhythmic syndromes is possible, but a diagnosis has first to be made. Given the proarrhythmic events explained above, which occur in patients with diabetes, an argument presents itself for obtaining a family history for young sudden death or arrhythmic syndromes, and a 12 lead ECG in every case. We suggest that good quality DNA should be retained following sudden death of a young person with diabetes, as should be standard practice for any sudden unexplained natural death in the young [33, 34]. If a conclusive cause of death is not found, the family should be referred to a cardiac genetic service, and genetic screening of autopsy DNA for cardiac ion channelopathies, particularly Brugada syndrome, should be considered. A molecular genetic cause of death can be made from DNA even when a blood sample (such as stored on a Guthrie card) is over 30 years old [16].

## Figures and Tables

**Table 1:** *In  silico* analysis of *GPD1L* gene* mutation.

Protein prediction programmes								Splice site prediction programmes	
PolyPhen-2^a,∗∗^	Mutation Taster^b^	Mutation Assessor^c ^	I-MUTANT 3.0^d^	MutPred^e^	SNPs and GO^f^	SIFT^g^	SNAP^h^	ASSP^i^ Neural Network^j^ ESE^k^	HSF^l^
Benign (scores: 0.000; 0.001)	Disease-causing (*P*: 0.999)	Neutral	Neutral (RI: 5)	Deleterious (Pdel: 0.89)	Neutral (RI: 9)	Tolerated (SR: 1.0)	Neutral (RI: 2.0)	No effect	SRp55 site created SRp40 site broken

*Refseq transcript accession number NM_015141.3.3; Refseq protein accession number NP_055956.1; uniprot accession number Q8N335.

**Scores relate to predictions based on HumDiv and HumVar models.

^a^ http://genetics.bwh.harvard.edu/pph2/.

^b^ http://www.mutationtaster.org/;  *P* refers to the probability value, which is the probability of the prediction.

^c^ http://mutationassessor.org/.

^d^ http://gpcr2.biocomp.unibo.it/cgi/predictors/I-Mutant3.0/I-Mutant3.0.cgi; RI refers to reliability index.

^e^ http://mutpred.mutdb.org/; Pdel refers to the probability that the variant is a deleterious mutation.

^f^ http://snps-and-go.biocomp.unibo.it/snps-and-go/; RI refers to reliability index.

^g^ http://sift.jcvi.org/www/SIFT_BLink_submit.html; SR refers to a SeqRep value that corresponds to the fraction of sequences that contain one of the basic amino acids. A low fraction indicates the position is either severely gapped or unalignable and has little information, so it offers poor predictive capability. *P*  refers to the scaled probability that the amino acid change is deleterious (a value less than 0.05 is predicted to be deleterious).

^h^ https://rostlab.org/services/snap/submit; RI refers to reliability index.

^i^ http://wangcomputing.com/assp/index.html.

^j^ http://www.fruitfly.org/seq_tools/splice.html.

^k^ http://rulai.cshl.edu/cgi-bin/tools/ESE3/esefinder.cgi; screened for human U2-type splice donor and acceptor sites above default thresholds.

^l^ http://www.umd.be/HSF/.

## Abbreviations

CTGF: Gene encoding for connective tissue growth factor
G6PC: Gene encoding for glucose-6-phosphatase
GPD1L: Gene glycerol-3-phosphate dehydrogenase 1-like protein
PHOX2B: Gene encoding for paired mesoderm homeobox protein 2b
SCN5A: Gene encoding for sodium channel protein type 5 subunit alpha, isoform a
Na_v_1.5: Sodium ion channel protein, voltage-gated, type V, alpha subunit.
